# The impact of antidiabetic drugs on dementia risk: a Bayesian network meta-analysis

**DOI:** 10.3389/fendo.2026.1780676

**Published:** 2026-04-15

**Authors:** Yang Yu, Xun Peng, Chang Su, Yangguang Bai, Dan Hou

**Affiliations:** 1 Department of Radiation Oncology, The First Hospital of Jilin University, Changchun, China; 2 Department of Pathology and Lab Medicine, Shandong Cancer Hospital and Institute, Shandong First Medical University and Shandong Academy of Medical Sciences, Jinan, China; 3 College of Pharmacy, Dalian Medical University, Dalian, China; 4 Department of Pharmacy and Shandong Provincial Key Traditional Chinese Medical Discipline of Clinical Chinese Pharmacy, Shandong Cancer Hospital and Institute, Shandong First Medical University and Shandong Academy of Medical Sciences, Jinan, China

**Keywords:** Alzheimer’s disease, antidiabetic drugs, Bayesian network analysis, dementia risk, meta-analysis, systematic review

## Abstract

**Background:**

Diabetes is significantly associated with cognitive impairment, particularly the risk of developing dementia. However, the impact of antidiabetic drugs on dementia risk remains unclear. This study aims to comprehensively evaluate the effects of different antidiabetic drugs on dementia risk using Bayesian network analysis.

**Methods:**

The study systematically searched databases including PubMed, Embase, and the Cochrane Library to identify relevant publications up to September 5, 2025. Eligible randomized controlled trials, cohort studies, and case-control studies were selected. We employed a Bayesian network meta-analysis model to quantitatively assess the relationship between antidiabetic drugs and dementia risk. Data analysis was performed using R version 4.4.1.

**Results:**

A total of 28 articles (involving 4,382,897 patients), network meta-analysis results indicates that compared with placebo, Insulin [OR = 0.11, 95% CrI (0.1, 0.12)], Metformin [OR = 0.79, 95% CrI (0.77, 0.81)], and Pioglitazone [OR = 0.69, 95% CrI (0.56, 0.86)] all reduced the incidence of dementia compared to placebo, a higher incidence of Alzheimer’s dementia[OR = 1.78, 95% CrI (1.66, 1.91)] and Vascular dementia[OR = 2.59, 95% CrI (2.33, 2.88)] with DPP4i compared to SGLT_2i.

**Conclusion:**

This study indicate that insulin demonstrates the most pronounced efficacy in reducing the incidence risk of dementia and vascular dementia. Furthermore, SGLT_2i and GLP1 exhibit certain therapeutic benefits in the management of Alzheimer’s disease.

**Systematic review registration:**

https://www.crd.york.ac.uk/prospero/, identifier CRD420251172386.

## Background

Diabetes, particularly type 2 diabetes, has become one of the major global public health challenges (1). According to the World Health Organization, the number of people with diabetes worldwide continues to rise, with projections indicating that by 2030, the global diabetic population will reach 580 million (2). Diabetes not only causes abnormal blood glucose levels but also affects multiple organ systems, including the cardiovascular, renal, and ocular systems (3). A growing body of research indicates a strong association between diabetes and the risk of cognitive impairment, particularly dementia (4). With the advent of an aging society, the risk of dementia among diabetic patients has significantly increased, placing immense pressure on public health systems and medical resources (5, 6).

Dementia is a syndrome characterized by cognitive dysfunction arising from multiple causes (7). Its primary features include deterioration in memory, language, thinking, and executive functions, severely impairing patients’ ability to perform daily activities. Alzheimer’s disease is the most prevalent form of dementia, accounting for approximately 60% to 70% of all cases (8). Studies indicate that individuals with diabetes face a substantially higher risk of developing cognitive impairment or dementia compared to the general population (9, 10). From a biological perspective, several mechanisms support the plausibility of this association. Insulin resistance may impair insulin signaling in the brain, which is essential for neuronal survival, synaptic plasticity, and memory formation (11). Chronic hyperglycemia and insulin resistance can also induce systemic and neuroinflammation, increase oxidative stress, and promote endothelial dysfunction, ultimately leading to cerebrovascular injury and reduced cerebral perfusion (12). Moreover, diabetes-related vascular pathology accelerates small vessel disease, which plays a critical role in the development of vascular dementia and mixed dementia. This risk is particularly pronounced among those with type 2 diabetes, where persistent hyperglycemia, insulin resistance, metabolic disorders, and multiple vascular pathologies collectively heighten the likelihood of cognitive decline. The impact of diabetes on the brain can be explained through multiple mechanisms, including hyperglycemia-induced neurotoxicity, cerebrovascular injury, oxidative stress, and inflammatory responses (13). Prolonged hyperglycemia may cause functional impairment of cerebral blood vessels, inadequate blood supply, and neuronal degeneration, thereby accelerating the onset of dementia (14).

As the number of diabetes patients increases, drug therapy has become a key approach for controlling blood glucose and slowing the progression of diabetic complications. Common antidiabetic medications include oral drugs and insulin (15). In recent years, with the continuous introduction of novel antidiabetic drugs such as GLP1receptor agonists, DPP4i, and SGLT_2i, their therapeutic effects have gradually gained recognition (16). However, the impact of different antidiabetic drugs on cognitive function remains highly controversial. Research indicates that long-term use of certain antidiabetic drugs (insulin and sulfonylureas) may be associated with cognitive decline, particularly in elderly patients (17). Side effects of these medications—such as hypoglycemic episodes and weight gain—can exacerbate cognitive deterioration. Additionally, blood glucose fluctuations represent an independent risk factor that may accelerate cognitive impairment. Conversely, some newer antidiabetic drugs, such as GLP1receptor agonists and SGLT_2i, have demonstrated neuroprotective effects (18). GLP1receptor agonists, by stimulating insulin secretion, suppressing appetite, and exerting anti-inflammatory effects, have been shown in animal models to improve cognitive function and slow dementia progression (19). SGLT_2i lower blood glucose levels by reducing renal glucose reabsorption, and some clinical studies suggest these drugs may offer brain protection in diabetic patients (20). Nevertheless, existing research findings remain inconsistent, and no clear consensus exists on whether these medications significantly reduce dementia risk in individuals with diabetes (21, 22). Therefore, this study employs a Bayesian network meta-analysis to systematically evaluate the impact of different antidiabetic drugs on dementia risk. It aims to reveal the potential role of diabetes medications in cognitive health, provide more scientific evidence for clinical practice, and offer new insights for cognitive health management in diabetic patients.

## Methods

This systematic evaluation and meta-analysis will strictly follow the PRISMA (Preferred Reporting Items for Systematic Reviews and Meta-Analyses) guidelines (23).

And it is registered in Prospero with registration number CRD420251172386.

### Literature search

This study searched PubMed, Embase, and the Cochrane Library, with a cutoff date of September 5, 2025. Search terms included “diabetes mellitus,” “antidiabetic drugs,” “cognitive decline,” “dementia,” and “Alzheimer’s disease.” The specific search strategy is detailed in **Supplementary Material Supplementary Table 1**.

### Inclusion criteria

The studies included in this research must meet the following criteria: participants must be diabetic patients with a confirmed diagnosis of type 2 diabetes or type 1 diabetes; the studies must include treatment with at least one antidiabetic medication, which may include oral hypoglycemic agents (such as sulfonylureas, metformin, GLP1, SGLT_2i) and/or insulin therapy; The primary objective of this network meta-analysis was to evaluate the association between antidiabetic medication use and the risk of overall dementia. Secondary objectives included examining the associations with specific dementia subtypes, namely Alzheimer’s disease and vascular dementia, when data were available. Most studies ascertained outcomes using standardized diagnostic systems, including the Diagnostic and Statistical Manual of Mental Disorders (DSM-IV or DSM-5) or the International Classification of Diseases (ICD-9 or ICD-10), derived from medical records, hospital discharge diagnoses, or national health registries. In cohort studies based on administrative databases, dementia diagnoses were primarily identified using ICD codes, while randomized controlled trials and some cohort studies applied clinical diagnostic assessments. When multiple diagnostic approaches were reported, priority was given to clinician-confirmed diagnoses. Study designs must be randomized controlled trials, cohort studies, or case-control studies, and must be original research.

### Exclusion criteria

Excluded studies must meet the following conditions: studies involving non-diabetic subjects or those without a confirmed diagnosis of diabetes; studies evaluating only the impact of antidiabetic drugs on glycemic control without addressing cognitive function or dementia; non-original research such as review articles, meta-analyses, conference abstracts, expert opinions, and case reports; studies failing to report changes in cognitive function, dementia incidence, or Alzheimer’s disease diagnoses.

### Data extractions

Two authors independently screened the literature for inclusion by importing the literature into endnote according to the literature inclusion and exclusion criteria, the final included studies were used for data extraction using excel software and if there was a dispute about the literature screening then it would be discussed, or a third person would be sought to adjudicate. The extracted data contained basic characteristics of the study (first author, year of publication, country, study design), basic characteristics of the population (sample size, gender, mean age), intervention, and outcome.

### Risk of bias

For randomized controlled trials, two researchers independently assessed risk of bias using the Cochrane Collaboration’s tools (24). If there was any disagreement, a third person was consulted to reach agreement. The assessment included seven areas: generation of randomized sequences (selective bias), allocation concealment (selective bias), blinding of implementers, blinding of outcome assessors (observational bias), completeness of outcome data (follow-up bias), and other potential sources of bias. Each study was individually examined against these criteria. When all criteria were fully met, the study was at ‘low risk’ of bias and was of high quality. Where some of the criteria were met, the quality was ‘unclear risk’, i.e. there was a moderate possibility of bias. When a study did not fulfil any of the criteria, it was classified as ‘high risk’, indicating a low-quality study. For cohort studies, and case-control studies, the Newcastle Ottawa Scale (25) will be used to assess quality. The NOS evaluates studies based on three dimensions: population selection, comparability, and exposure or outcome, with eight items totaling nine points. Scores range from 0 to 4 (low quality), 5 to 6 (moderate quality), and 7 to 9 (high quality). Studies scoring 0 to 4 will be excluded.

### Statistical analysis

In this study, statistical analysis will be performed using a Bayesian framework for Network Meta-Analysis (NMA) (26) to compare the efficacy of antidiabetic drugs on the risk of dementia, a treatment network will be constructed by connecting studies that directly or indirectly compare two or more treatments. Each treatment will serve as a node, and edges between nodes represent direct comparisons between treatments performed in individual studies. Bayesian network meta-analysis will then be conducted. For dichotomous outcomes, effect sizes will be expressed as odds ratios (ORs) with corresponding 95% credible intervals (CrIs), whereas mean difference (MD) will be used for continuous outcomes. A Bayesian approach allows the incorporation of prior distributions and the use of Markov chain Monte Carlo (MCMC) methods to generate posterior distributions of treatment effects.

Non-informative (vague) prior distributions will be assigned to treatment effect parameters, while a minimally informative prior will be used for the between-study heterogeneity parameter to reduce undue influence on posterior estimates. To account for inter-study heterogeneity, a random-effects model will be applied throughout the analysis.

Model convergence will be assessed using trace plots, density plots, and the Gelman–Rubin potential scale reduction factor (PSRF), with values close to 1.0 indicating adequate convergence. A burn-in period will be discarded prior to formal sampling to ensure model stability. In this study, Deviance Information Criterion (DIC) will be used to assess the consistency of the network Meta-analysis model. Specifically, two models will be constructed: one assuming consistency and the other assuming inconsistency. By comparing the DIC values of these two models, we can determine whether there is inconsistency in the network. To account for inter-study heterogeneity, a random effects model will be used for the analysis. Heterogeneity will be assessed by the I² statistic, with significant heterogeneity indicated if the I² value exceeds 50%. Network consistency will also be tested to ensure consistency between direct and indirect evidence. If inconsistency is found, possible causes will be explored, and the model will be adjusted through sensitivity analysis. All statistical analyses will be performed using R software (version 4.4.1) and the “gemtc” package, which is specifically designed for Bayesian network meta-analysis. The results will be presented in the form of a posteriori means, confidence intervals (95% CrI) and ranking probabilities, thus providing reliable conclusions on the comparison of treatments.

To address heterogeneity arising from differences in study design, a random-effects Bayesian network meta-analysis model was applied throughout the analysis. Formal subgroup analyses by study design were not performed, as stratification would compromise the connectivity of the treatment network.

## Results

### Literature retrieval results

**Figure 1:**
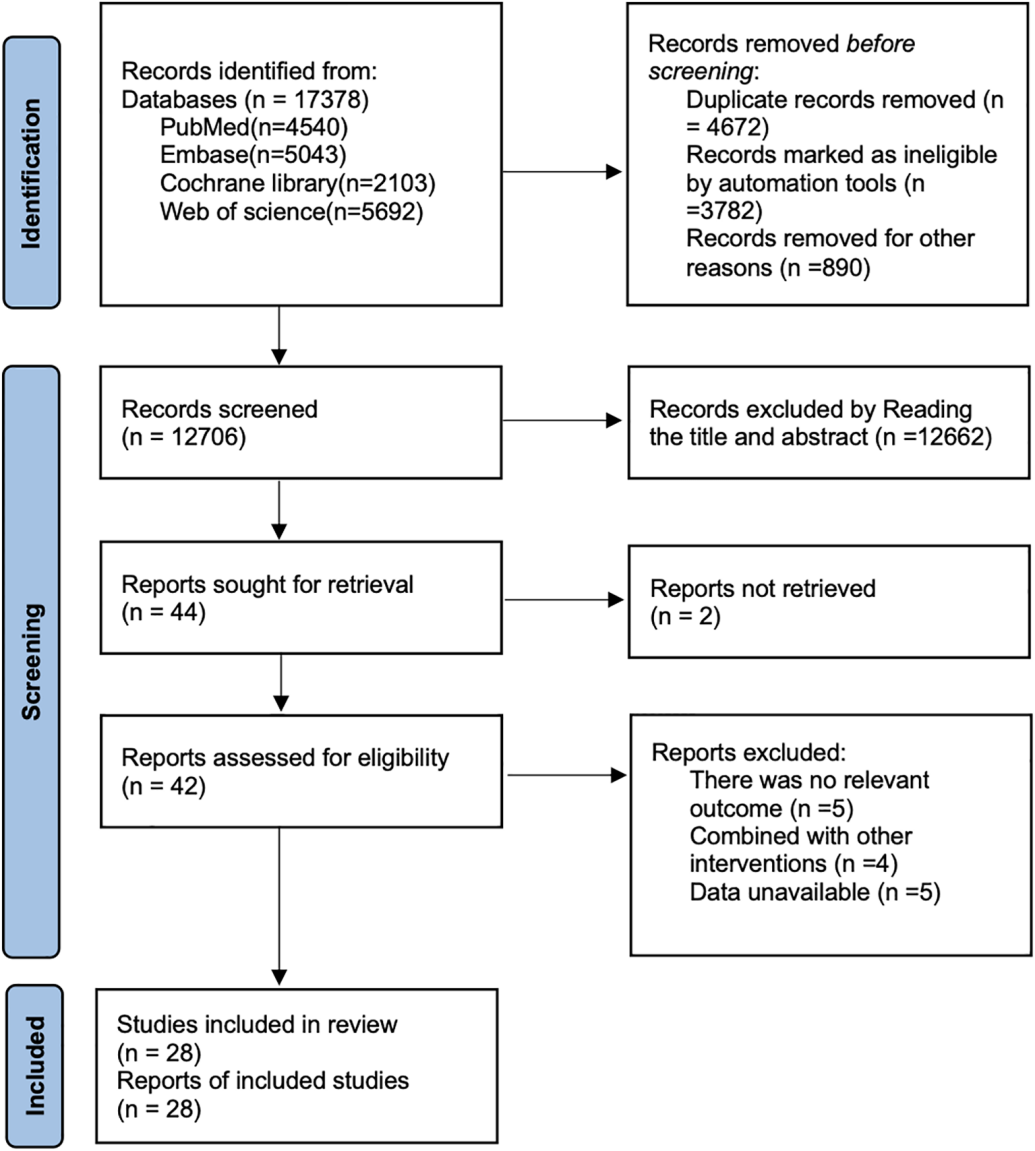
Literature search flow chart.

As shown in **Figure 1**, a total of 17,378 articles were retrieved from PubMed (n=4,540), Embase (n=5,043), the Cochrane Library (n=2,103), Web of Science (n=5692), a total of 17,378 articles were identified. After removing 4,672 duplicate records, 12,662 articles were excluded based on title and abstract screening. Two articles were inaccessible for full-text retrieval, and 14 articles were excluded after full-text review. Ultimately, 28 articles (27–54) were included for analysis.

### Table of basic characteristics

This study included a total of 28 articles (involving 4,382,897 patients with diabetes), comprising 1 randomized controlled trial, 1 case-control study, and 26 cohort studies. The average age was 64.23 years, with most studies (64.3%) originating from Asia (China, South Korea, Japan). Specific baseline characteristics are presented in **Table 1**.

**Table 1:** Table of basic characteristics of the literature.

Study	Year	Country	Study design	Sample size	Gender(M/F)	Mean age(years)	Intervention	outcomes
Abdullah	2025	Canada	cohort study	SGLT-2i:34816 DPP-4i:83190	77150/40856	>40	SGLT-2i DPP-4i	F1; F2; F3; F4
Alkabbani	2023	Canada	cohort study	Insulin:7863 Placebo:25230	15647/17446	57.32	Insulin	F1
Biessels	2019	Netherlands	Randomized controlled study	DPP-4i:800 Placebo:745	1004/541	67.8	DPP-4i	F1
Chen	2020	China	cohort study	DPP-4i:2903 Placebo:11612	5545/8970	68.03	DPP-4i	F1
Chen	2023	China	cohort study	Metformin:31384 Placebo:31384	35338/27430	55.22	Metformin	F1
Cheng	2025	China	cohort study	GLP1:54960 Placebo:439680	253476/241164	65.2	GLP1	F1
Chin	2019	China	cohort study	Metformin:15676 Placebo:15676	17994/13358	63.5	Metformin	F1
Chou	2017	China	cohort study	Pioglitazone:6401 Placebo:12802	9513/9690	65.1	Pioglitazone	F1
Cukierman	2020	Israel	cohort study	GLP1:4456 Placebo:4372	4720/4108	65.5	dulaglutide	F2
Ha	2021	Korea	Case-control	Metformin:8921 Placebo:1130	5641/4410	65	Metformin	F3
Hong	2024	Korea	cohort study	SGLT-2i:2076 GLP1:1038	1589/1525	66.8	SGLT-2i; dulaglutide	F1; F3; F4
Hong	2024	Korea	cohort study	SGLT-2i:42873 DPP-4i:384757	177112/250518	59.8	SGLT-2i; DPP-4i	F1
Hou	2025	China	cohort study	Metformin:2031 Placebo:16866	8863/10034	60.8	Metformin	F1
Hsu	2011	China	cohort study	Metformin:1864 Sulfonylureas:3753 Placebo:10519	8206/7930	64.3	Metformin; Sulfonylureas	F1
Inoue	2025	Japan	cohort study	GLP1:2418 DPP-4i:4836	3261/3993	64.1	GLP1; DPP-4	F1
Kim	2020	Korea	cohort study	Metformin:3632 Placebo:4436	4093/3975	64.1	Metformin	F1
Kuan	2017	China	cohort study	Metformin:4651 Placebo:4651	4695/4607	63.2	Metformin	F1; F3; F4
Liu	2025	China	cohort study	SGLT-2i:80376 DPP-4i:80376	86274/68946	69.7	SGLT-2i; DPP-4i	F1; F3; F4
Ng	2014	Singapore	cohort study	Metformin:204 Placebo:161	151/214	67	Metformin	F2
Orkaby	2017	USA	cohort study	Metformin:17200 Sulfonylureas:11440	28314/326	66	Metformin; Sulfonylureas	F1; F3; F4
Shi	2019	USA	cohort study	Metformin:2774 Placebo:2756	5408/122	63.24	Metformin	F1; F2; F3
Shin	2024	Korea	cohort study	SGLT-2i:110885 DPP-4i:110885	123538/98232	64.3	SGLT-2i; DPP-4i	F1; F3; F4
Sun	2024	China	cohort study	Metformin:7279 Placebo:7279	7279/7279	66.5	Metformin	F1
Tang	2024	Sweden	cohort study	GLP1:12351 DPP-4i:43850 Sulfonylureas:32216	48960/39457	66.1	GLP1; DPP-4i; Sulfonylureas	F1
Tseng	2020	China	cohort study	Acarbose:15524 Placebo:15524	16828/14220	67.8	Acarbose	F1
Wang	2025	USA	cohort study	GLP1:64267 Insulin:1156564	1145371/75460	61.4	GLP1; Insulin	F1; F4
Wu	2023	Canada	cohort study	SGLT-2i:36513 DPP-4i:36545	44657/28401	62.4	SGLT-2i; DPP-4i	F1
Zheng	2023	UK	cohort study	Metformin:114628 Placebo:95609	114678/95559	61.9	Metformin	F1

F1, Dementia; F2, mild cognitive impairment; F3, Alzheimer dementia; F4, Vascular dementia.

### Risk of bias results

For the quality assessment results of randomized controlled trials (**Supplementary Material Supplementary Figure 1**), the study clearly described the randomization method used, thus rated as low risk. It also described the blinding method, rated as low risk. For case-control and cohort studies (**Supplementary Material Supplementary Table 2**), the overall scores ranged from 7 to 9 points, indicating that the included articles demonstrated high overall quality.

### Pairwise meta-analysis

This study employed a Pairwise meta-analysis. The results (**Table 2**) indicate that for dementia, SGLT_2i versus DPP_4_ inhibitors [OR = 0.42, 95% CrI (0.25, 0.691)], insulin *vs* GLP1[OR = 0.09, 95% CrI (0.03, 0.28)], and sulfonylureas *vs* GLP1[OR = 3.63, 95% CrI (1.19, 11)]. For mild cognitive impairment, Alzheimer dementia, and vascular dementia, no significant differences were observed in the Pairwise meta-analysis.

**Table 2:** Pairwise meta-analysis results.

Outcomes	Pairwise meta-analysis	No of study	Heterogeneity (%)	OR 95%CrI
Dementia	Acarbose VS Placebo	1	NA	1.14(0.382, 3.45)
	GLP1 vs DPP 4i	2	89.2	0.65(0.29, 1.45)
	Placebo vs DPP_4i	2	89.6	0.96(0.43, 2.13)
	SGLT_2i vs DPP_4i	5	99.3	0.42(0.25, 0.691)
	Sulfonylureas vs DPP_4i	1	NA	1.90(0.629, 5.46)
	Insulin vs GLP1	1	NA	0.09(0.03, 0.28)
	Placebo vs GLP1	1	NA	0.93(0.31, 2.83)
	SGLT_2i vs GLP1	1	NA	0.80(0.25,2.59)
	Sulfonylureas vs GLP1	1	NA	3.63(1.19, 11)
	Placebo vs Insulin	1	NA	0.72(0.24, 2.20)
	Placebo vs Metformin	9	98.8	1.10(0.76, 1.60)
	Sulfonylureas vs Metformin	2	97.4	1.78(0.79, 3.96)
	Placebo vs Pioglitazone	1	NA	1.44(0.48, 4.41)
	Sulfonylureas vs Placebo	1	NA	1.19(0.36, 5.90)
Outcomes	Pairwise meta-analysis	No of study	Heterogeneity (%)	OR 95%CrI
Mild cognitive impairment	Placebo vs GLP1	1	NA	1.08(0.53, 2.23)
	Placebo vs Metformin	2	0	0.69(0.29, 1.62)
Outcomes	Pairwise meta-analysis	No of study	Heterogeneity (%)	OR 95%CrI
Alzheimer dementia	SGLT_2i vs DPP_4i	3	98.7	0.44(0.15, 1.17)
	SGLT_2i vs GLP1	1	NA	0.78(0.13, 4.62)
Outcomes	Pairwise meta-analysis	No of study	Heterogeneity (%)	OR 95%CrI
Vascular dementia	SGLT_2i vs DPP_4i	3	97.4	0.28(0.04, 1.78)
	Insulin vs GLP1	1	NA	0.11(0.01, 2.60)
	SGLT_2i vs GLP1	1	NA	2.78(0.06, 127)

### Results of consistency modeling

The current study used a random-effects model to compare the difference in DIC between consistent and inconsistent modeling, and the absolute value of the difference in DIC was <5, The results (**Supplementary Material Supplementary Table 3**) indicate that dementia, mild cognitive impairment, Alzheimer dementia and vascular dementia has consistency.

### Dementia

**Figure 2:**
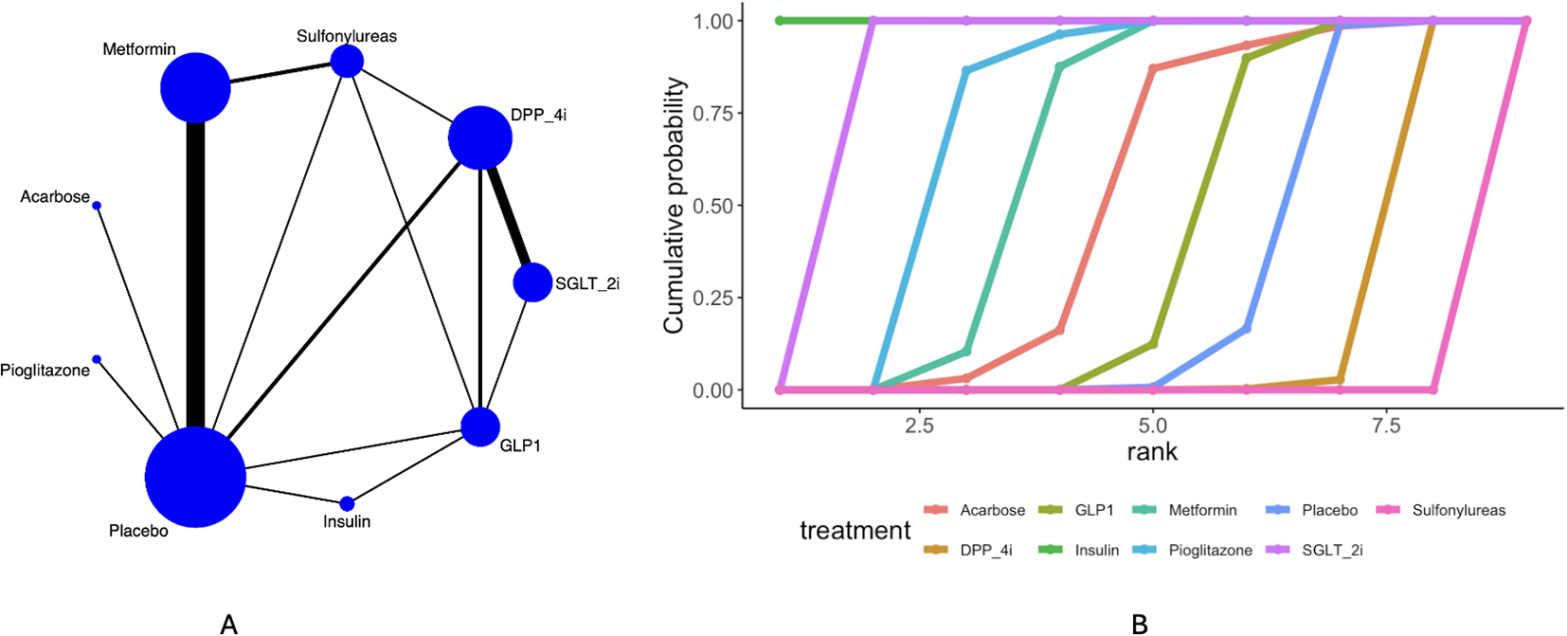
Results of network meta-analysis of dementia **(A)** network diagram, **(B)** cumulative probability ranking diagram.

Twenty-four articles mentioned dementia. The network diagram (**Figure 2A**) indicated the formation of a closed loop, prompting a local inconsistency test. Results (**Supplementary Materials Supplementary Figure 2**) revealed direct and indirect comparisons showing inconsistencies in the following pairs: GLP1*vs* DPP_4i, Placebo *vs* DPP_4i, SGLT-2i *vs* DPP_4i, Sulfonylureas *vs* DPP_4i, Sulfonylureas *vs* Metformin, Sulfonylureas *vs* Placebo, Placebo *vs* GLP-1, SGLT-2i *vs* GLP-1, Sulfonylureas *vs* GLP-1, and Placebo *vs* Metformin showed no significant differences between direct, indirect, and network comparisons. Statistically significant differences existed between direct, indirect, and network comparisons for Insulin *vs* GLP1and Placebo *vs* Insulin. The league table (**Supplementary Material Supplementary Table 4**) indicates that compared with placebo, Insulin [OR = 0.11, 95% CrI (0.1, 0.12)], Metformin [OR = 0.79, 95% CrI (0.77, 0.81)], and Pioglitazone [OR = 0.69, 95% CrI (0.56, 0.86)] all reduced the incidence of dementia compared to placebo. Insulin demonstrated superior efficacy compared to Metformin [OR = 0.14, 95% CrI (0.12, 0.15)] and Pioglitazone [OR = 0.16, 95% CrI (0.12, 0.2)]. Curve cumulative probability ranking (**Figure 2B**, **Table 3**) revealed the following order: Insulin (99.9%) > SGLT_2i (87.5%) > Pioglitazone (72.9%) > Sulfonylureas (0.01%).

**Table 3:** Sucar rank.

Treatment	Dementia (%)	Alzheimer dementia(%)	Vascular dementia (%)
Acarbose	49.7	NR	NR
DPP_4i	12.9	2.6	1.20
GLP1	37.8	52.9	58.8
Insulin	99.9	NR	99.9
Metformin	62.3	NR	NR
Pioglitazone	72.9	NR	NR
Placebo	26.9	NR	NR
SGLT_2i	87.5	94.4	39.9
Sulfonylureas	0.01	NR	NR

### Mild cognitive impairment

**Figure 3:**
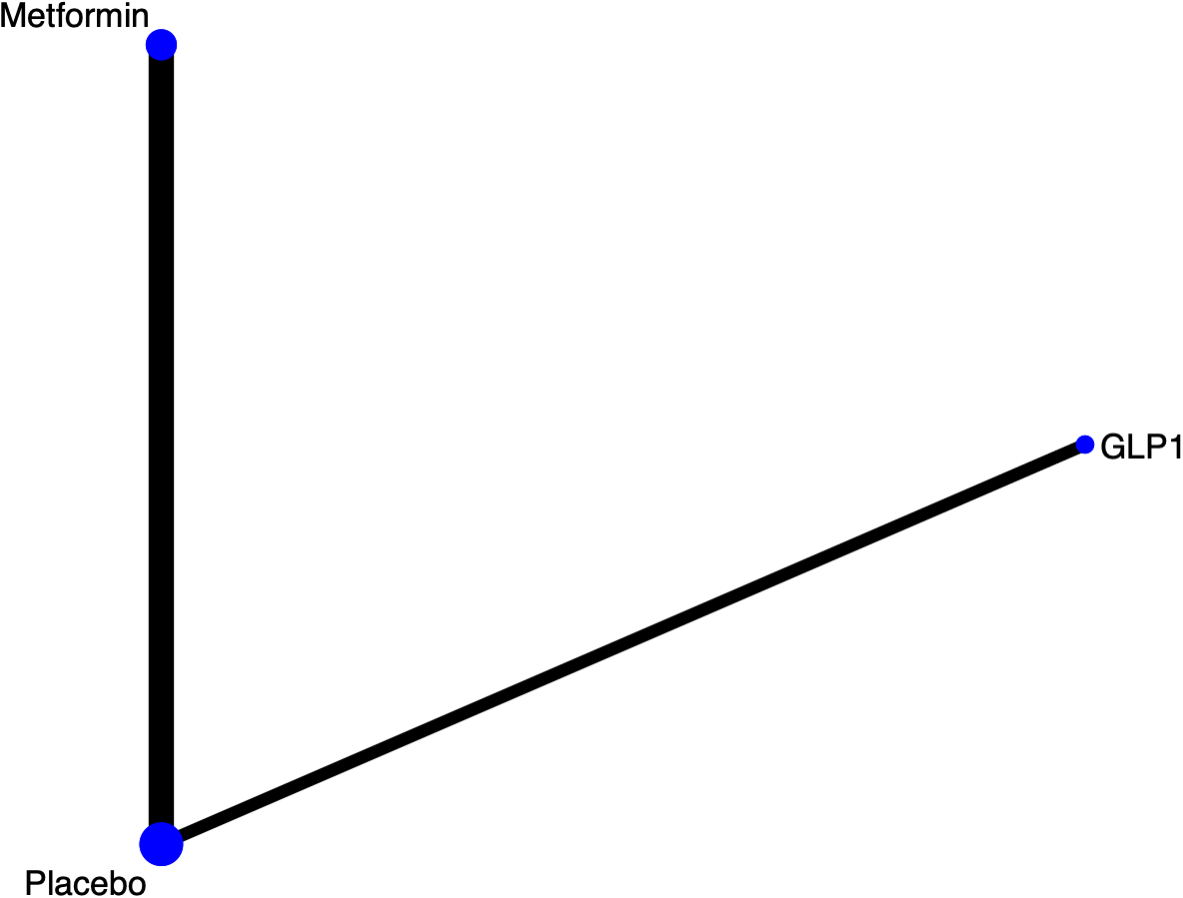
Results of network meta-analysis of Mild cognitive impairment network diagram.

Three studies mentioned mild cognitive impairment. The network diagram (**Figure 3**) indicates direct comparisons between Metformin and GLP1versus placebo. The league table (**Supplementary Material Supplementary Table 5**) suggests no significant differences between different intervention comparisons. Therefore, this outcome measure is not ranked in this analysis.

### Alzheimer dementia

**Figure 4:**
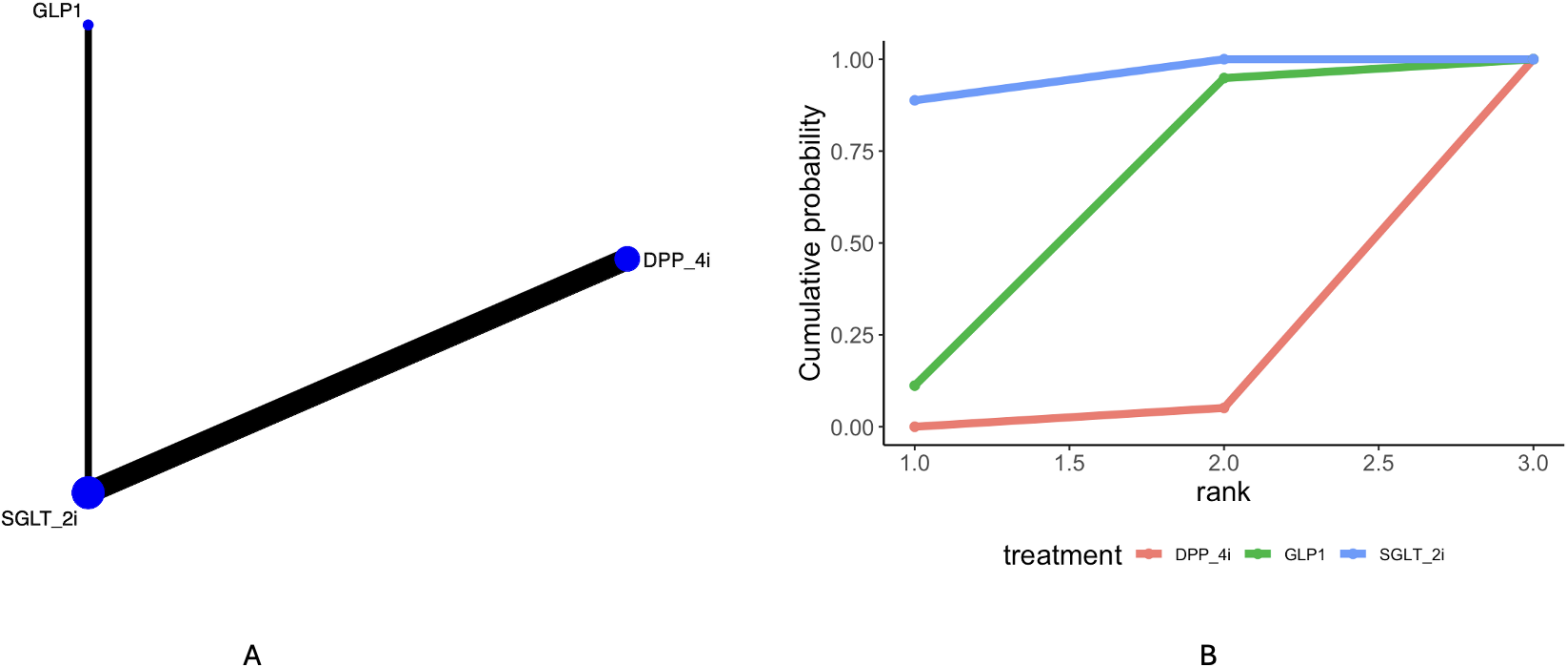
Results of network meta-analysis of Alzheimer dementia **(A)** network diagram, **(B)** cumulative probability ranking diagram.

Four articles mentioned Alzheimer’s dementia. The network plot (**Figure 4A**) indicates direct comparisons between SGLT_2i, DPP4i, and GLP1. The league table (**Supplementary Material Supplementary Table 6**) suggests a higher incidence of Alzheimer’s dementia with DPP4i compared to SGLT_2i [OR = 1.78, 95% CrI (1.66, 1.91)]. Ranking by cumulative probability curves (**Figure 4B**, **Table 3**) indicates SGLT_2i (94.4%) > GLP1(52.9%) > DPP4i (2.6%).

### Vascular dementia

**Figure 5:**
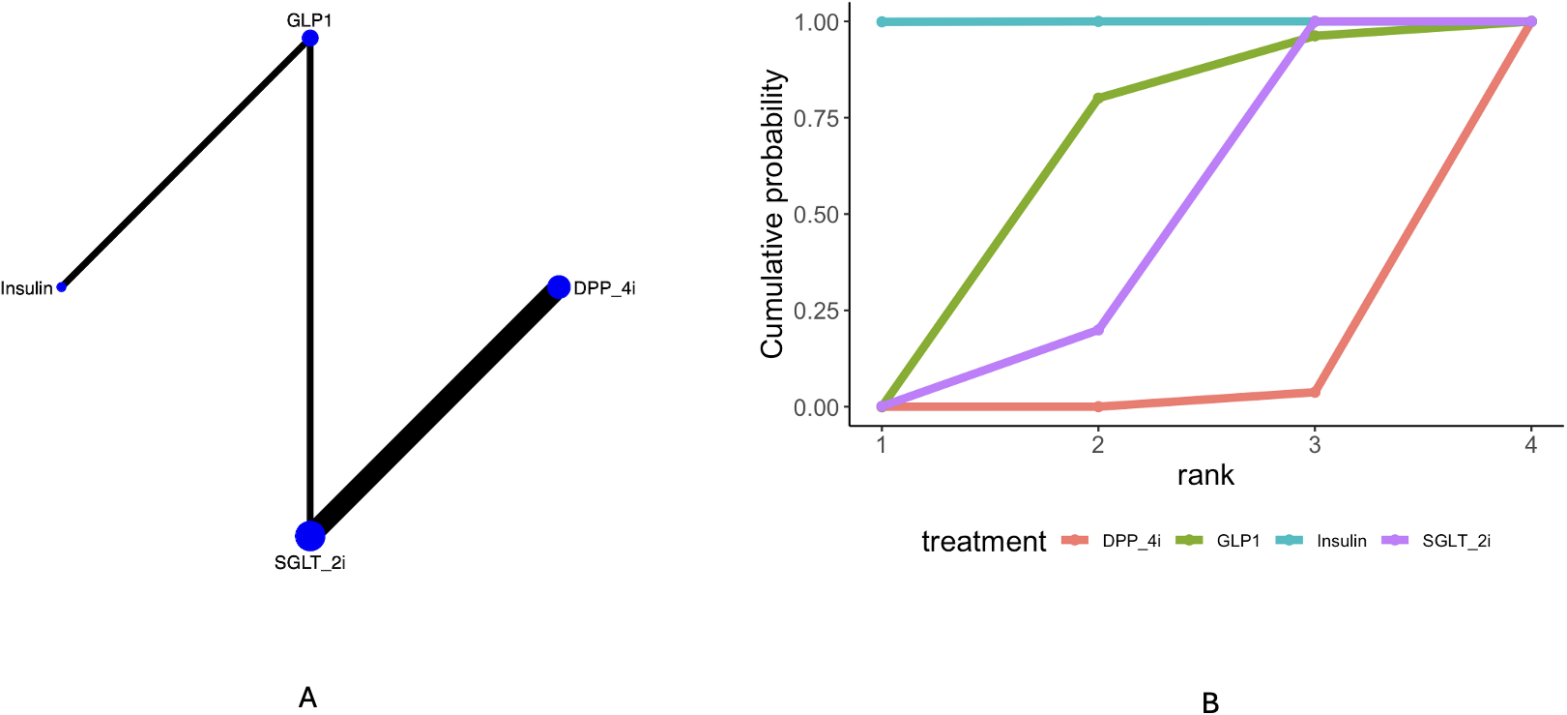
Results of network meta-analysis of vascular dementia **(A)** network diagram, **(B)** cumulative probability ranking diagram.

Five articles mentioned Vascular dementia. The network plot (**Figure 5A**) indicates direct comparisons between SGLT_2i, DPP4i, Insulin and GLP1agonists. The league table (**Supplementary Material Supplementary Table 7**) suggests a higher incidence of Vascular dementia with DPP4i compared to SGLT_2i [OR = 2.59, 95% CrI (2.33, 2.88)]. Ranking by cumulative probability curves (**Figure 5B**, **Table 3**) indicates Insulin (99.9%) > GLP1(58.8%) > SGLT_2i (39.9%).

### Publication bias

This study assessed publication bias using funnel plots. The results (**Supplementary Materials Supplementary Figures 3**-**6**) suggest that the likelihood of publication bias is low for dementia, but higher for mild cognitive impairment, Alzheimer’s dementia, and vascular dementia.

## Discussion

This study employed a Bayesian network meta-analysis to evaluate the impact of various antidiabetic drugs on the risk of dementia and its subtypes (mild cognitive impairment, Alzheimer’s disease, and vascular dementia). Results indicated insulin demonstrated the greatest efficacy in reducing dementia risk. Compared with placebo, insulin, metformin, and pioglitazone all effectively lowered dementia incidence. For Alzheimer’s disease, SGLT_2i exhibited superior therapeutic efficacy. In vascular dementia treatment, insulin likewise demonstrated optimal efficacy. The comparison between DPP-4 inhibitors and SGLT-2 inhibitors should be interpreted with caution. Although the network meta-analysis suggested a higher incidence of Alzheimer’s disease and vascular dementia associated with DPP-4 inhibitors, these estimates were derived largely from indirect evidence. Indirect comparisons are inherently dependent on the assumption of transitivity and consistency across the network and may be influenced by differences in study populations, baseline risk, and comparator treatments (55). Consequently, the certainty of evidence for this comparison is lower than for comparisons supported by robust direct evidence. Future head-to-head randomized or well-designed comparative observational studies are needed to strengthen confidence in these findings.

The exceptionally large protective effect observed for insulin (OR = 0.11) should be interpreted with caution. Several factors may have contributed to this magnitude of effect. First, residual confounding cannot be fully excluded, particularly in observational studies, where insulin users may differ systematically from non-users in disease duration, glycemic control, healthcare access, and clinical monitoring intensity. Second, indication bias may have influenced the results, as insulin is often prescribed to patients with more frequent medical follow-up, potentially increasing the likelihood of early detection and management of cognitive impairment. Third, population differences across studies, including age distribution, comorbidity profiles, and baseline dementia risk, may have amplified observed effect estimates.

Moreover, insulin exposure was often treated as a binary variable in the included studies, without accounting for dosage, treatment duration, or glycemic variability, which may further overestimate its apparent protective effect. Therefore, although insulin demonstrated the strongest association with reduced dementia risk in the network meta-analysis, this finding should not be interpreted as definitive evidence of a causal or uniformly large clinical benefit. Whilst metformin and pioglitazone exhibited less pronounced effects than insulin, their results remained clinically significant and possess considerable therapeutic potential. It is worth noting, however, that while these drugs demonstrate statistically significant differences, their clinical significance may be limited. For instance, metformin exhibited an (OR = 0.79) compared to placebo. Although this effect is statistically significant, its practical clinical impact may be insufficient to produce widespread effects across diabetic populations. For clinicians, such modest effect changes are unlikely to substantially alter treatment decisions, particularly when alternative therapeutic options exist.

SGLT_2i may exert indirect protective effects on brain health by lowering blood glucose and improving cardiovascular health. The mechanism of action for GLP1 agonists appears more indirect, primarily functioning through the improvement of metabolic abnormalities and cardiovascular health (56). Both SGLT_2i and GLP1 have demonstrated statistical significance in the treatment of Alzheimer’s disease, though their actual clinical impact may fall short of expectations (57). The cumulative probability for SGLT_2i stands at 94.4%, indicating their relatively prominent efficacy in network analysis. However, this does not imply such pronounced effects will be achieved in all clinical practice settings. Indeed, the actual efficacy of medications is frequently influenced by patient variability, drug tolerance, and other comorbidities. Consequently, despite these drugs performing well in statistical analyses, their practical application necessitates comprehensive consideration of individual patient circumstances to avoid blind reliance on statistical significance (58). Particularly regarding DPP_4i, although research indicates a higher incidence of Alzheimer’s disease (OR = 1.78), the clinical significance of this finding warrants further evaluation. Although DPP_4i use may increase dementia risk, this finding carries not exclusively negative clinical implications, as these agents retain advantages in glycemic control and mitigating other diabetic complications (59). Clinicians should thus conduct holistic assessments considering patients’ overall health status and potential adverse effects, rather than relying solely on isolated metrics.

In the treatment of vascular dementia, insulin has demonstrated the most potent efficacy. This finding supports the hypothesis that insulin may slow the progression of vascular dementia in diabetic patients by protecting cerebral vascular function and reducing cerebral vascular lesions (60). Insulin not only improves glucose metabolism but may also exert beneficial effects on the cerebral vascular system through mechanisms such as its anti-inflammatory properties and promotion of vasodilation (61, 62). However, despite insulin’s significant efficacy against vascular dementia, some research findings suggest that long-term insulin use may be associated with certain side effects, particularly concerning cardiovascular health in diabetic patients (63). Although insulin demonstrated superiority in network meta-analyses, its clinical application requires caution. Crucially, alongside glycemic control, effectively mitigating potential adverse effects—such as weight gain and hypoglycemia—remains a significant consideration in clinical decision-making.

Potential misclassification bias should be considered when interpreting these findings. Dementia subtypes, particularly Alzheimer’s disease and vascular dementia, may be misclassified across studies due to differences in diagnostic criteria, data sources, and clinical practice. Studies relying on administrative databases and ICD codes may underdiagnose or misclassify dementia subtypes, especially in early or mixed presentations. Such non-differential misclassification could bias effect estimates toward the null and partially explain inconsistencies across comparisons. Despite this limitation, the use of standardized diagnostic systems in most included studies and the consistency observed in network analyses support the overall robustness of the findings. Nevertheless, future studies using uniform diagnostic criteria and adjudicated outcomes are warranted.

### Strengths and limitations

The strengths of this study lie in its large sample size and diverse research designs. The inclusion of 28 studies encompassing 4,382,897 diabetic patients provides substantial sample numbers, enhancing the representativeness and reliability of the analytical findings. Furthermore, the variety of research designs—including randomized controlled trials, case-control studies, and cohort studies—broadens the applicability of conclusions, circumventing the limitations inherent in single-design studies. The application of Bayesian network meta-analysis enabled simultaneous comparisons of different drugs, circumventing the sequential nature of traditional analyses and providing more comprehensive evidence. Furthermore, consistency modelling analysis was employed to validate the robustness of findings, ensuring coherence across statistical models and thereby enhancing the reliability of the study’s conclusions.

Despite the multiple strengths of this study, several limitations should be acknowledged.

First, most of the included studies were observational in design, which inherently limits causal inference and may introduce residual confounding, even after statistical adjustment. Unmeasured or inadequately controlled factors could not be fully excluded.

Second, substantial variability existed in follow-up duration across studies, which may have influenced the assessment of cognitive outcomes and limited the comparability of results, particularly with respect to long-term cognitive trajectories.

Third, although this review encompassed multiple types of cognitive impairment, data concerning mild cognitive impairment were relatively scarce, and the corresponding estimates demonstrated considerable uncertainty, thereby reducing the robustness and generalizability of conclusions in this subgroup.

Moreover, the lack of long-term longitudinal studies precluded a comprehensive evaluation of the sustained effects of antidiabetic medications on cognitive decline.

Finally, although publication bias was assessed, the potential omission of unpublished or negative findings cannot be entirely ruled out, which may have affected the overall comprehensiveness of the conclusions, especially in domains such as mild cognitive impairment and Alzheimer’s disease.

### Future research directions

Future research requires the conduct of additional long-term, prospective randomized controlled trials to validate the sustained protective effects of these medications against cognitive decline, particularly in diabetic populations. Furthermore, forthcoming studies should investigate therapeutic variations across distinct cohorts—including differing age groups, genders, and comorbidities—to advance the development of personalized treatment regimens. Finally, further investigation into the potential mechanisms by which antidiabetic drugs improve cognitive function—particularly their roles in insulin resistance, neuroprotection, and cardiovascular health—is essential to better understand the rationale for their clinical application.

## Conclusion

This study employed a Bayesian network meta-analysis to comprehensively evaluate the associations between different antidiabetic medications and the risk of dementia and its subtypes, including mild cognitive impairment, Alzheimer’s disease, and vascular dementia. The findings suggest that insulin was associated with the most pronounced reduction in the incidence of overall dementia and vascular dementia, while SGLT-2 inhibitors and GLP-1 receptor agonists, primarily driven by evidence from specific agents such as liraglutide and exenatide, were associated with a reduced risk of dementia.

From a clinical perspective, these results may provide supportive evidence for considering the potential cognitive effects of antidiabetic medications when selecting glucose-lowering therapies, particularly in patients with diabetes who are at increased risk of cognitive decline. However, the observed effect sizes—especially the substantial protective association observed with insulin—should be interpreted cautiously due to the possibility of confounding factors, indication bias, and differences in patient populations across studies. Moreover, some statistically significant findings may not translate into clinically meaningful benefits, underscoring the importance of individualized risk–benefit assessment.

In clinical decision-making, the choice of antidiabetic therapy should remain primarily guided by glycemic control needs, cardiovascular and renal comorbidities, safety profiles, and patient preferences. The potential cognitive benefits of SGLT-2 inhibitors and GLP-1 receptor agonists may represent an additional consideration, rather than a primary indication, when tailoring treatment strategies for patients with long-term diabetes or elevated dementia risk. Future well-designed randomized controlled trials and prospective studies are warranted to clarify causal relationships, assess long-term cognitive outcomes, and further inform evidence-based clinical practice.

## Data Availability

The original contributions presented in the study are included in the article/**Supplementary Material**. Further inquiries can be directed to the corresponding author.
