# Supplementary figures and images for: The impact of antidiabetic drugs on dementia risk: a Bayesian network meta-analysis
Source: Front Endocrinol (Lausanne). 2026 Apr 15;17:1780676. doi: 10.3389/fendo.2026.1780676 (PMC13124527; doi:10.3389/fendo.2026.1780676)

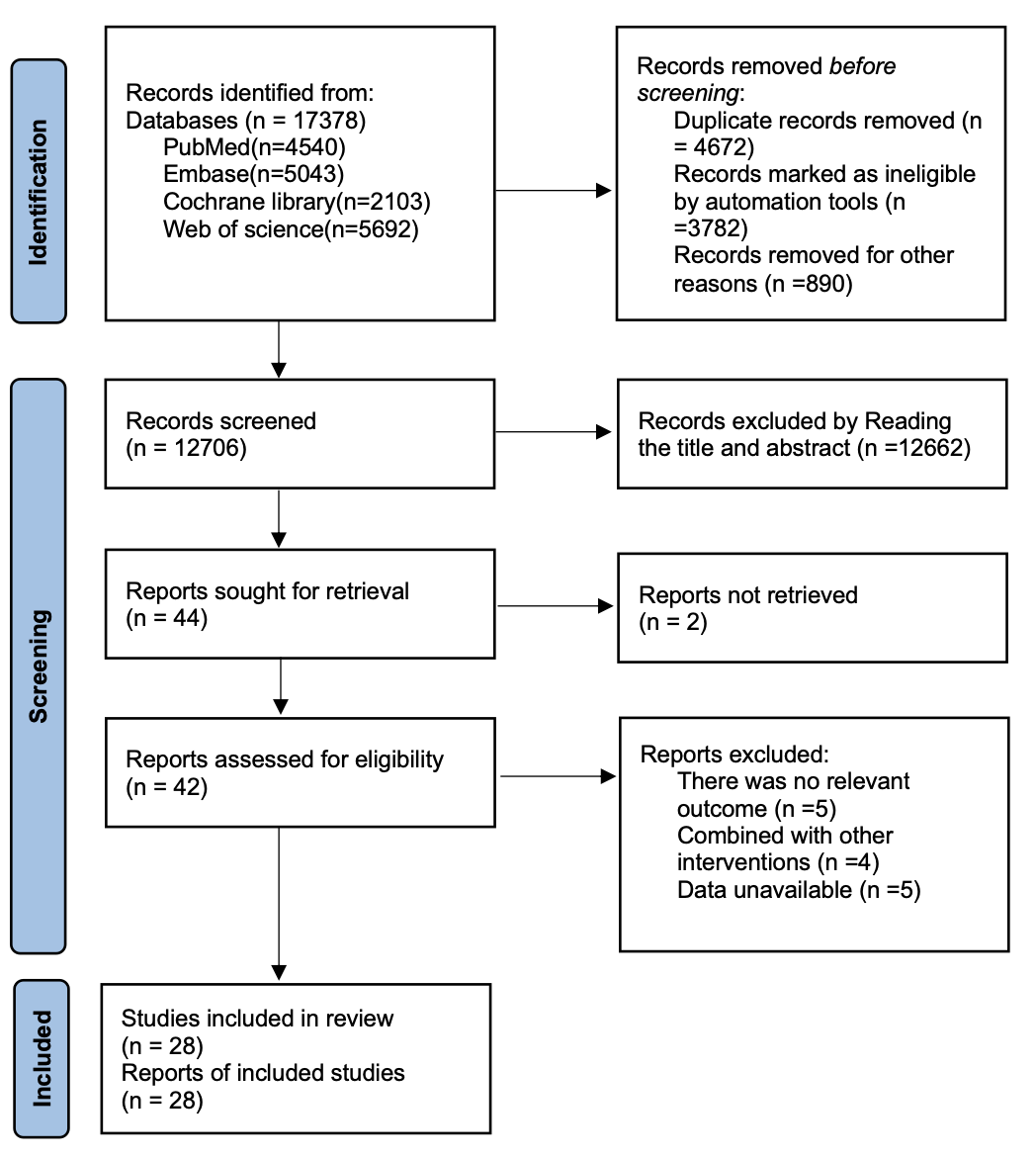

Supplement: Supplementary file 1 [file Image1.png]

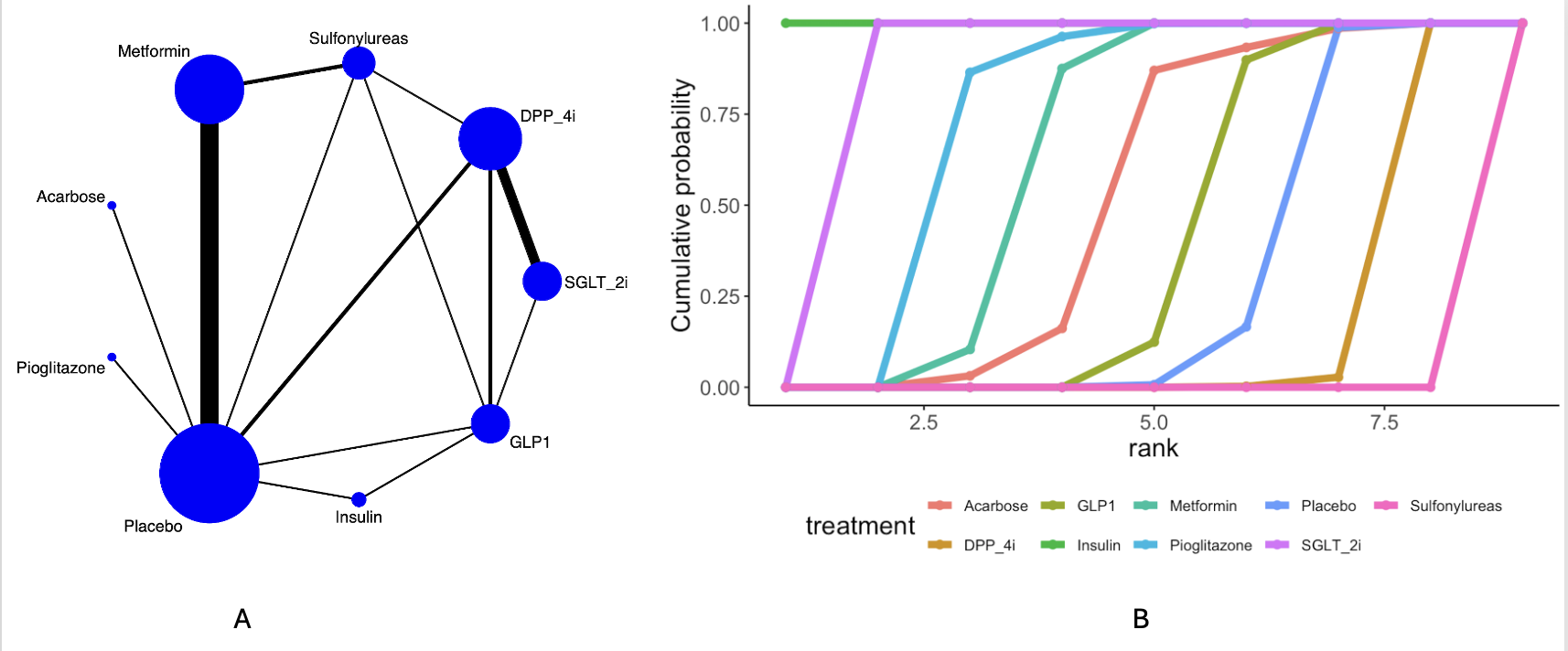

Supplement: Supplementary file 2 [file Image2.png]

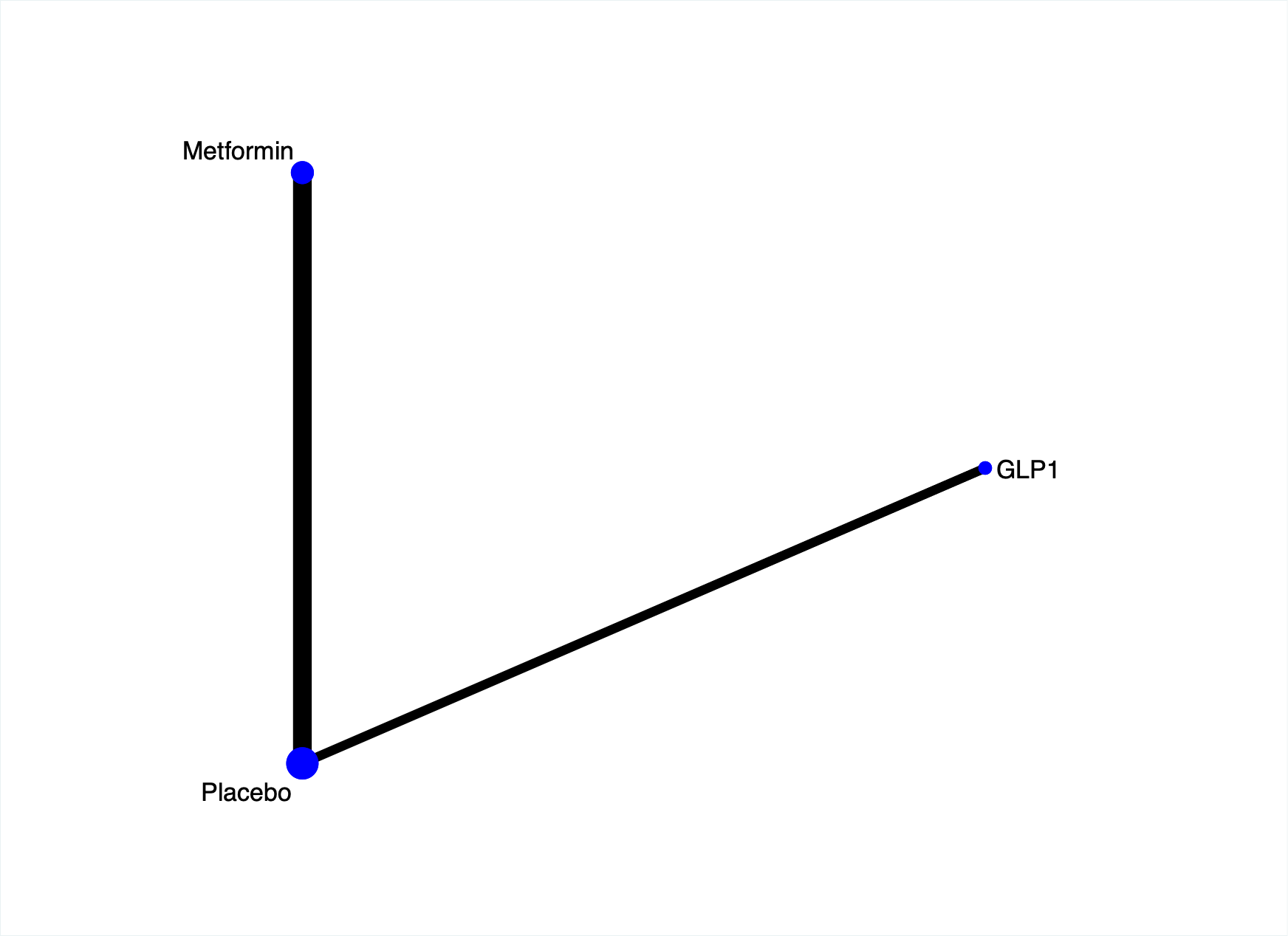

Supplement: Supplementary file 3 [file Image3.png]

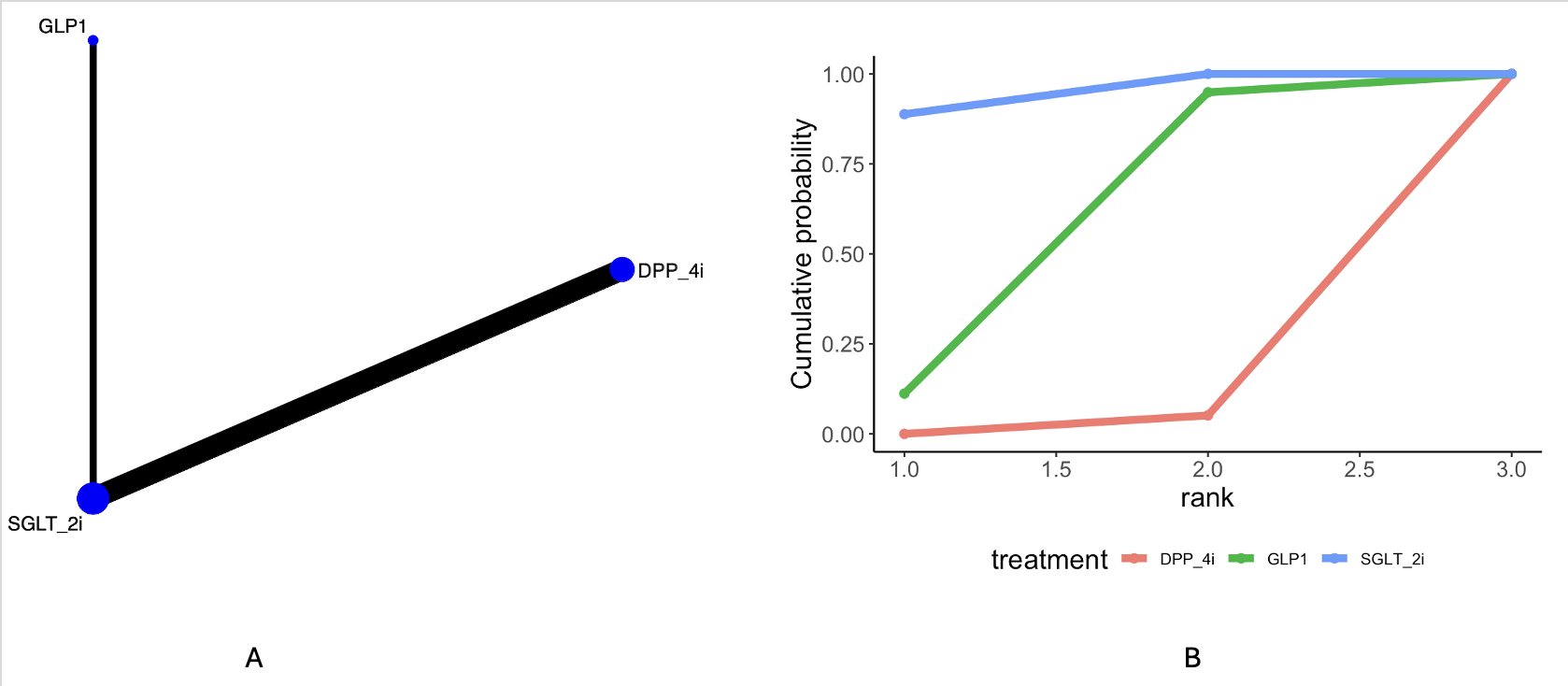

Supplement: Supplementary file 4 [file Image4.png]

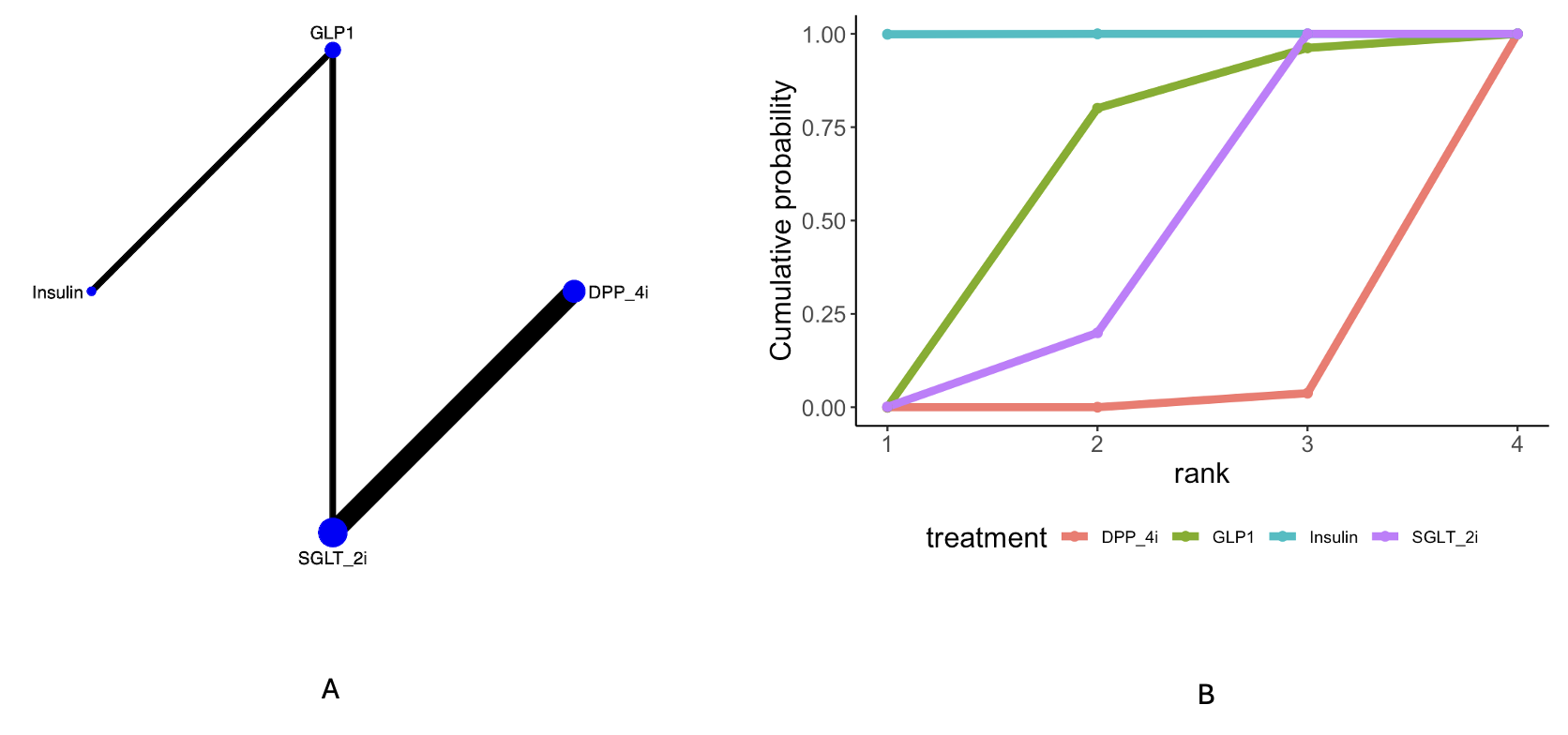

Supplement: Supplementary file 5 [file Image5.png]
