# Supplementary material for: The impact of antidiabetic drugs on dementia risk: a Bayesian network meta-analysis
Source: Front Endocrinol (Lausanne). 2026 Apr 15;17:1780676. doi: 10.3389/fendo.2026.1780676 (PMC13124527; doi:10.3389/fendo.2026.1780676)
Supplement: Supplementary file 6 [file Table1.docx]

Table S1 specific search strategy

(("Dementia"[Mesh]) OR (((((((((((((((Dementia[Title/Abstract]) OR (Dementias[Title/Abstract])) OR (Amentia[Title/Abstract])) OR (Amentias[Title/Abstract])) OR (Senile Paranoid Dementia[Title/Abstract])) OR (Dementias, Senile Paranoid[Title/Abstract])) OR (Paranoid Dementia, Senile[Title/Abstract])) OR (Paranoid Dementias, Senile[Title/Abstract])) OR (Senile Paranoid Dementias[Title/Abstract])) OR (Familial Dementia[Title/Abstract])) OR (Dementia, Familial[Title/Abstract])) OR (Dementias, Familial[Title/Abstract])) OR (Familial Dementias[Title/Abstract])) OR (Alzheimer Disease[Title/Abstract])) OR (Vascular dementia[Title/Abstract]))) AND (("Metformin"[Mesh]) OR ((((((((((((((((((((((((((((((((((((((((((((((((((((((((((((((((((((((((((((((((((((((((((((((Metformin[Title/Abstract]) OR (Dimethylbiguanidine[Title/Abstract])) OR (Dimethylguanylguanidine[Title/Abstract])) OR (Glucophage[Title/Abstract])) OR (Metformin Hydrochloride[Title/Abstract])) OR (Hydrochloride, Metformin[Title/Abstract])) OR (Metformin HCl[Title/Abstract])) OR (HCl, Metformin[Title/Abstract])) OR (Sulfonylurea Compounds[Title/Abstract])) OR (Sulfonylurea[Title/Abstract])) OR (Acetohexamide[Title/Abstract])) OR (Carbutamide[Title/Abstract])) OR (Chlorpropamide[Title/Abstract])) OR (Gliclazide[Title/Abstract])) OR (Glyburide[Title/Abstract])) OR (Tolazamide[Title/Abstract])) OR (Tolbutamide[Title/Abstract])) OR (Thiazolidinedione[Title/Abstract])) OR (Glitazones[Title/Abstract])) OR (Dipeptidyl-Peptidase IV Inhibitors[Title/Abstract])) OR (Dipeptidyl Peptidase IV Inhibitors[Title/Abstract])) OR (DPP-4 Inhibitor[Title/Abstract])) OR (DPP 4 Inhibitor[Title/Abstract])) OR (Inhibitor, DPP-4[Title/Abstract])) OR (DPP-IV Inhibitor[Title/Abstract])) OR (DPP IV Inhibitor[Title/Abstract])) OR (Inhibitor, DPP-IV[Title/Abstract])) OR (DPP-4 Inhibitors[Title/Abstract])) OR (DPP 4 Inhibitors[Title/Abstract])) OR (DPP-IV Inhibitors[Title/Abstract])) OR (DPP IV Inhibitors[Title/Abstract])) OR (DPP4 Inhibitors[Title/Abstract])) OR (Dipeptidyl Peptidase 4 Inhibitor[Title/Abstract])) OR (Dipeptidyl-Peptidase IV Inhibitor[Title/Abstract])) OR (Dipeptidyl Peptidase IV Inhibitor[Title/Abstract])) OR (Inhibitor, Dipeptidyl-Peptidase IV[Title/Abstract])) OR (Dipeptidyl-Peptidase 4 Inhibitor[Title/Abstract])) OR (Inhibitor, Dipeptidyl-Peptidase 4[Title/Abstract])) OR (Dipeptidyl-Peptidase 4 Inhibitors[Title/Abstract])) OR (Dipeptidyl Peptidase 4 Inhibitors[Title/Abstract])) OR (Gliptins[Title/Abstract])) OR (DPP4 Inhibitor[Title/Abstract])) OR (Gliptin[Title/Abstract])) OR (dipeptidyl peptidase-4(DPP-4[Title/Abstract]))) OR (Glycoside Hydrolase Inhibitors[Title/Abstract])) OR (Hydrolase Inhibitors, Glycoside[Title/Abstract])) OR (Inhibitors, Glycoside Hydrolase[Title/Abstract])) OR (alpha-Glucosidase Inhibitors[Title/Abstract])) OR (alpha Glucosidase Inhibitors[Title/Abstract])) OR (Inhibitors, alpha-Glucosidase[Title/Abstract])) OR (alpha-Glucosidase Inhibitor[Title/Abstract])) OR (alpha Glucosidase Inhibitor[Title/Abstract])) OR (Inhibitor, alpha-Glucosidase[Title/Abstract])) OR (Pancreatic alpha-Amylase Inhibitors[Title/Abstract])) OR (Inhibitors, Pancreatic alpha-Amylase[Title/Abstract])) OR (Pancreatic alpha Amylase Inhibitors[Title/Abstract])) OR (alpha-Amylase Inhibitors, Pancreatic[Title/Abstract])) OR (alpha Amylase Inhibitors, Pancreatic[Title/Abstract])) OR (Intestinal alpha-Amylase Inhibitors[Title/Abstract])) OR (alpha-Amylase Inhibitors, Intestinal[Title/Abstract])) OR (Inhibitors, Intestinal alpha-Amylase[Title/Abstract])) OR (Intestinal alpha Amylase Inhibitors[Title/Abstract])) OR (Glucagon-Like Peptide 1[Title/Abstract])) OR (Glucagon Like Peptide 1[Title/Abstract])) OR (GLP-1[Title/Abstract])) OR (GLP 1[Title/Abstract])) OR (Glucagon-Like Peptide-1[Title/Abstract])) OR (Liraglutide[Title/Abstract])) OR (Sodium-Glucose Transporter 2[Title/Abstract])) OR (Sodium Glucose Transporter 2[Title/Abstract])) OR (SGLT2 Protein[Title/Abstract])) OR (SLC5A2 Protein[Title/Abstract])) OR (Dapagliflozin[Title/Abstract])) OR (Empagliflozin[Title/Abstract])) OR (Canagliflozin[Title/Abstract])) OR (Ertugliflozin[Title/Abstract])) OR (Luseogliflozin[Title/Abstract])) OR (Ipragliflozin[Title/Abstract])) OR (Tofogliflozin[Title/Abstract])) OR (Remogliflozin[Title/Abstract])) OR (Sotagliflozin[Title/Abstract])) OR (insulin[Title/Abstract])) OR (Insulin, Regular[Title/Abstract])) OR (Regular Insulin[Title/Abstract])) OR (Soluble Insulin[Title/Abstract])) OR (Insulin, Soluble[Title/Abstract])) OR (Iletin[Title/Abstract])) OR (Insulin A Chain[Title/Abstract])) OR (Novolin[Title/Abstract])) OR (Sodium Insulin[Title/Abstract])) OR (Insulin, Sodium[Title/Abstract])) OR (Insulin B Chain[Title/Abstract])) OR (Chain, Insulin B[Title/Abstract])) OR (benzoic acid derivatives[Title/Abstract])))

Table S2 NOS results

| case control | | | | | | | | | |
| --- | --- | --- | --- | --- | --- | --- | --- | --- | --- |
| Study | Is the case definition adequate? | Representativeness of the cases | Determination of control group | Definition of Controls | Comparability of cases and controls based on the design or analysis | Ascertainment of exposure | Same method of ascertainment for cases and controls | Non response | Total scores |
| Ha2021 | * | * | * | * | ** | * | * | * | 9 |

| cohort study | | | | | | | | | |
| --- | --- | --- | --- | --- | --- | --- | --- | --- | --- |
| Study | Representativeness of the exposed group | Selection of non-exposed groups | Determination of exposure factors | Identification of outcome indicators not yet to be observed at study entry | Comparability of exposed and unexposed groups considered in design and statistical analysis | design and statistical analysis | Adequacy of the study's evaluation of the outcome | Adequacy of follow-up in exposed and unexposed groups | Total scores |
| Abdullah2025 | * | * | * | * | ** | * | * | * | 9 |
| Alkabbani2023 | * | * | * | * | ** | * | * | * | 9 |
| Chen2020 | * | * | * | * | ** | * | * | * | 9 |
| Chen2023 | * | * | * | * | * | * | * | * | 8 |
| Cheng2025 | * | * | * | * | * | * | * | * | 8 |
| Chin2019 | * | * | * | * | * | * | * | * | 8 |
| Chou2017 | * | * | / | ** | * | * | * | * | 8 |
| Cukierman2020 | * | * | / | ** | * | * | * | * | 8 |
| Hong2024 | * | * | * | ** | * | * | * | * | 9 |
| Hong2024 | * | * | * | ** | * | * | * | * | 9 |
| Hou2025 | * | * | * | ** | * | * | * | * | 9 |
| Hsu2011 | * | * | * | / | * | * | * | * | 7 |
| Inoue2025 | * | * | * | ** | * | * | * | * | 9 |
| Kim2020 | * | * | * | / | * | * | * | * | 7 |
| Kuan2017 | * | * | * | * | * | * | * | * | 8 |
| Liu2025 | * | * | / | ** | * | * | * | * | 8 |
| Ng2014 | * | * | * | * | * | * | * | * | 8 |
| Orkaby2017 | * | * | / | ** | * | * | * | * | 8 |
| Shi2019 | * | * | * | ** | * | * | * | * | 9 |
| Shin2024 | * | * | * | ** | * | * | * | * | 9 |
| Sun2024 | * | * | * | ** | * | * | * | * | 9 |
| Tang2024 | * | * | * | ** | * | * | * | * | 9 |
| Tseng2020 | * | * | * | / | * | * | * | * | 7 |
| Wang2025 | * | * | * | / | * | * | * | * | 7 |
| Wu2023 | * | * | * | ** | * | * | * | * | 9 |
| Zheng2023 | * | * | * | ** | * | * | * | * | 9 |

Table S3 Results of consistency modeling

| Outcomes | Consistency test | Inconsistency test | I^2^(%) |
| --- | --- | --- | --- |
| Dementia | 105.1 | 108.9 | 90 |
| Mild cognitive impairment | 10.2 | 10.5 | 13 |
| Alzheimer dementia | 118.2 | 119.8 | 98 |
| Vascular dementia | 107.71 | 110.02 | 78 |

Table S4 League table for Dementia

| OR 95%CrI | | | | | | | | |
| --- | --- | --- | --- | --- | --- | --- | --- | --- |
| Acarbose |  |  |  |  |  |  |  |  |
| 0.81 (0.67, 0.98)* | DPP_4i |  |  |  |  |  |  |  |
| 0.9 (0.75, 1.08) | 1.11 (1.04, 1.19*) | GLP1 |  |  |  |  |  |  |
| 8.02 (6.49, 9.89) * | 9.91 (8.78, 11.18) * | 8.92 (8.06, 9.85) * | Insulin |  |  |  |  |  |
| 1.11 (0.92, 1.32) | 1.37 (1.28, 1.46) * | 1.23 (1.17, 1.29) * | 0.14 (0.12, 0.15) * | Metformin |  |  |  |  |
| 1.26 (0.95, 1.67) | 1.56 (1.25, 1.96) * | 1.4 (1.13, 1.75) * | 0.16 (0.12, 0.2) * | 1.14 (0.92, 1.42) | Pioglitazone |  |  |  |
| 0.87 (0.73, 1.04) | 1.08 (1.01, 1.15) * | 0.97 (0.93, 1.02) | 0.11 (0.1, 0.12) * | 0.79 (0.77, 0.81) * | 0.69 (0.56, 0.86) * | Placebo |  |  |
| 2 (1.65, 2.42) * | 2.47 (2.39, 2.55) * | 2.22 (2.06, 2.4) * | 0.25 (0.22, 0.28) * | 1.81 (1.68, 1.95) * | 1.59 (1.26, 1.99) * | 2.29 (2.13, 2.46) * | SGLT_2i |  |
| 0.46 (0.38, 0.55) * | 0.57 (0.54, 0.6) * | 0.51 (0.48, 0.54) * | 0.06 (0.05, 0.06) * | 0.41 (0.39, 0.44) * | 0.36 (0.29, 0.45) * | 0.53 (0.5, 0.55) * | 0.23 (0.21, 0.24) * | Sulfonylureas |

* means P<0.05

Table s5 League table for Mild cognitive impairment

|  |  |  |
| --- | --- | --- |
| GLP1 |  |  |
| 0.63 (0.3, 1.29) | Metformin |  |
| 0.93 (0.84, 1.02) | 1.47 (0.72, 3.12) | Placebo |

Table s6 League table for Vascular Alzheimer dementia

| OR 95%CrI | | |
| --- | --- | --- |
| DPP_4i |  |  |
| 1.39 (0.93, 2.1) | GLP1 |  |
| 1.78 (1.66, 1.91)* | 1.28 (0.85, 1.89) | SGLT_2i |

* means P<0.05

Table s7 League table for Vascular dementia

| OR 95%CrI | | | |
| --- | --- | --- | --- |
| DPP_4i |  |  |  |
| 6.86 (0.86, 193.69) | GLP1 |  |  |
| 64.23 (7.89, 1810.05)* | 9.34 (7.61, 11.44) * | Insulin |  |
| 2.59 (2.33, 2.88) * | 0.38 (0.01, 3.02) | 0.04 (0, 0.33) * | SGLT_2i |

* means P<0.05

Figure s1 risk of bias summary


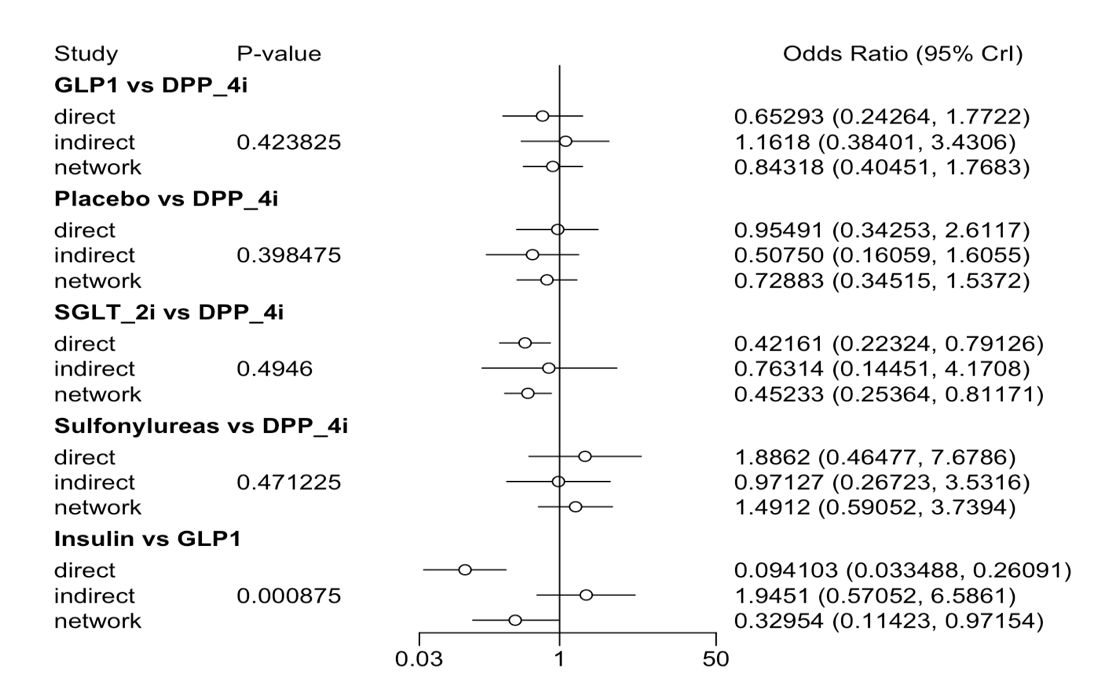


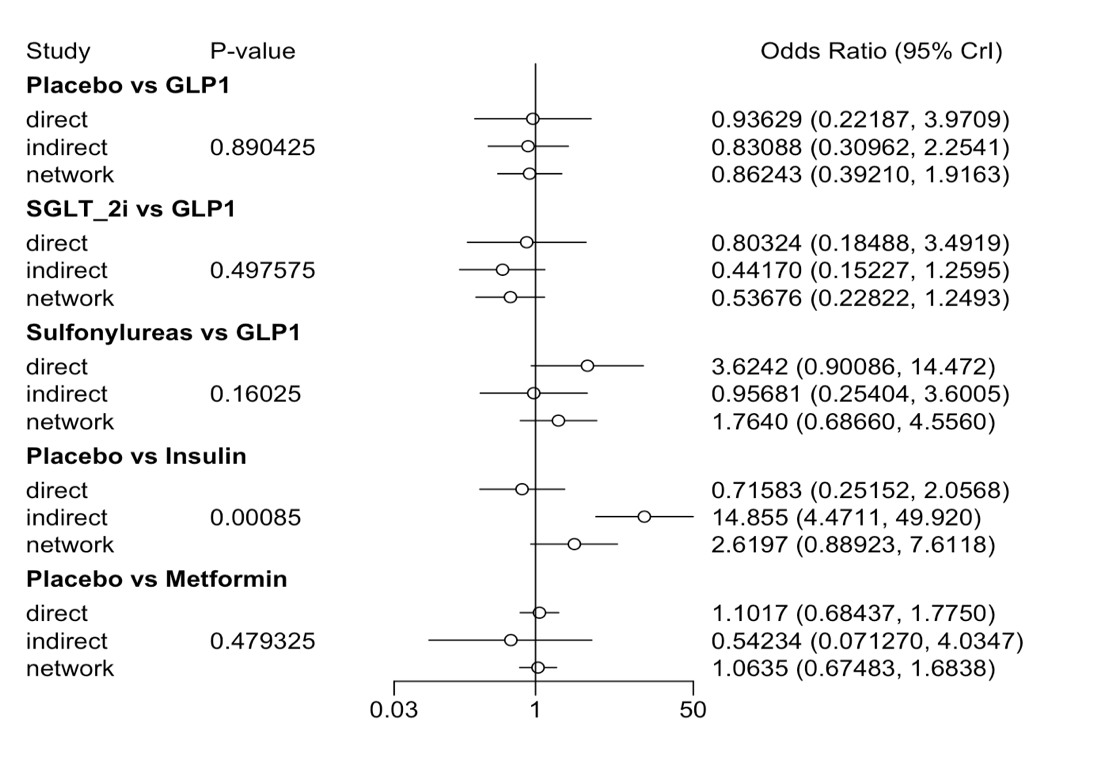


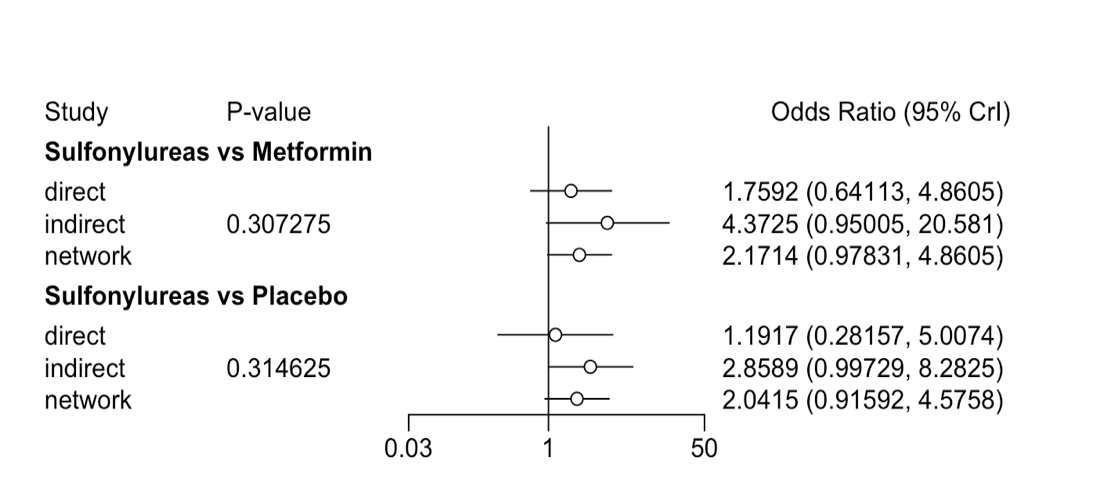


Figure s2 Local inconsistency test for dementia


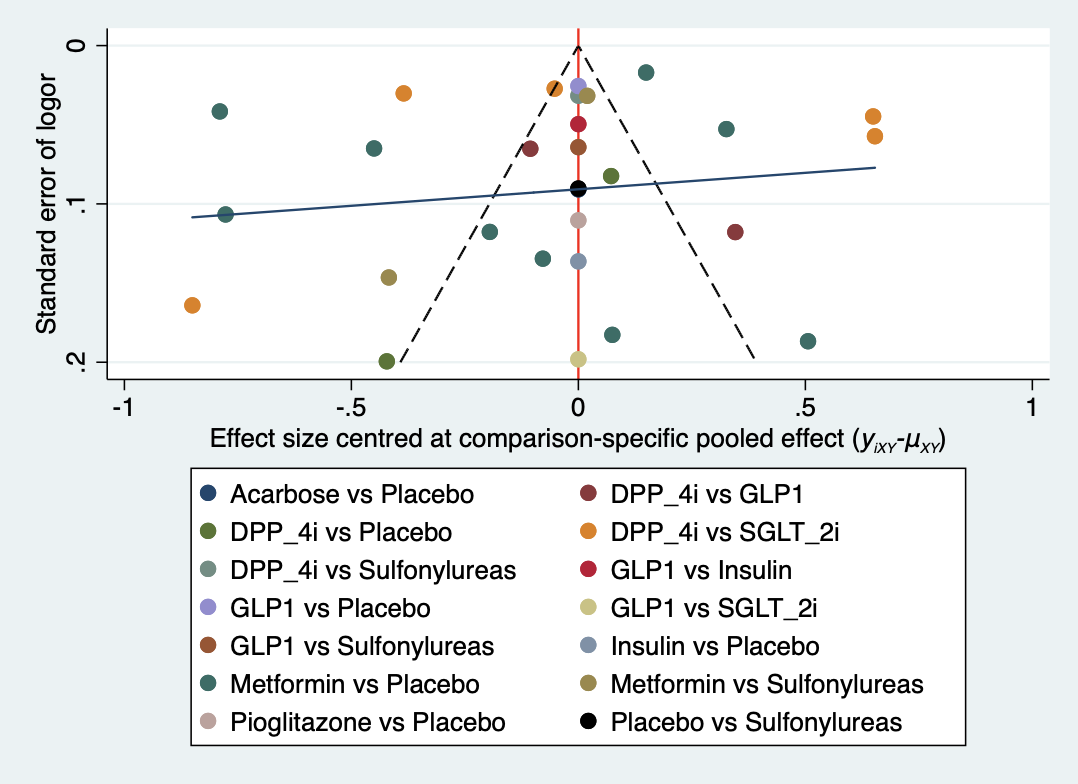

figure S3 Funnel plot of dementia


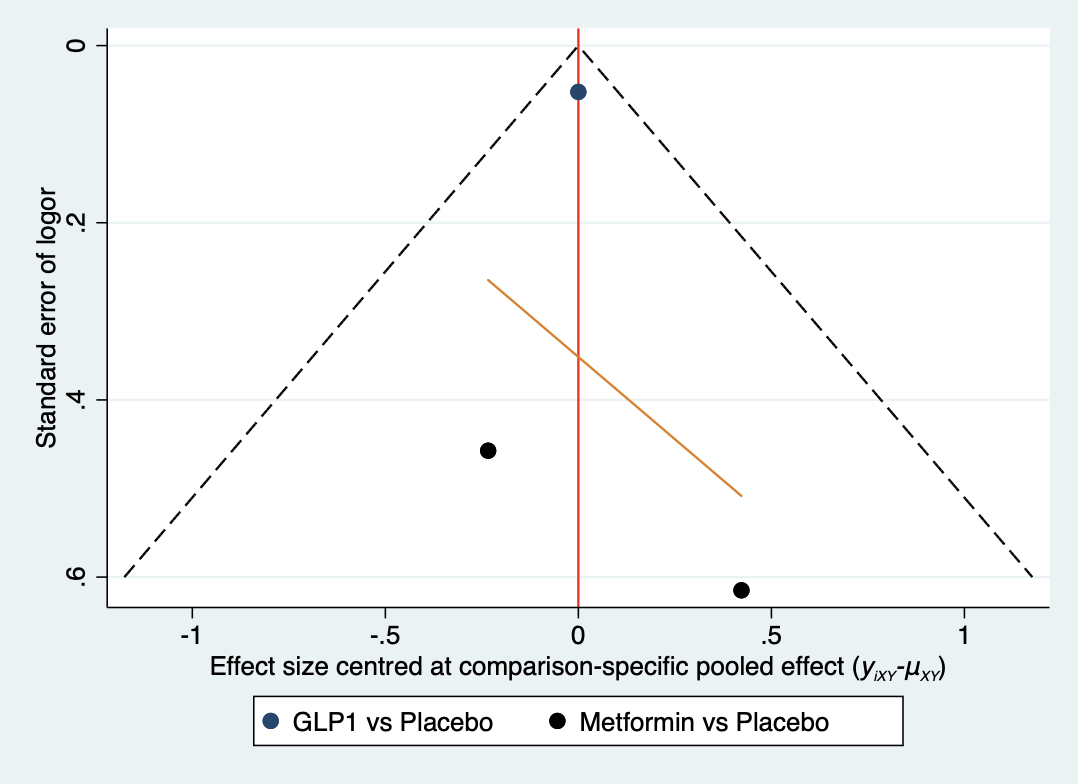


figure S4 Funnel plot of mild cognitive impairment


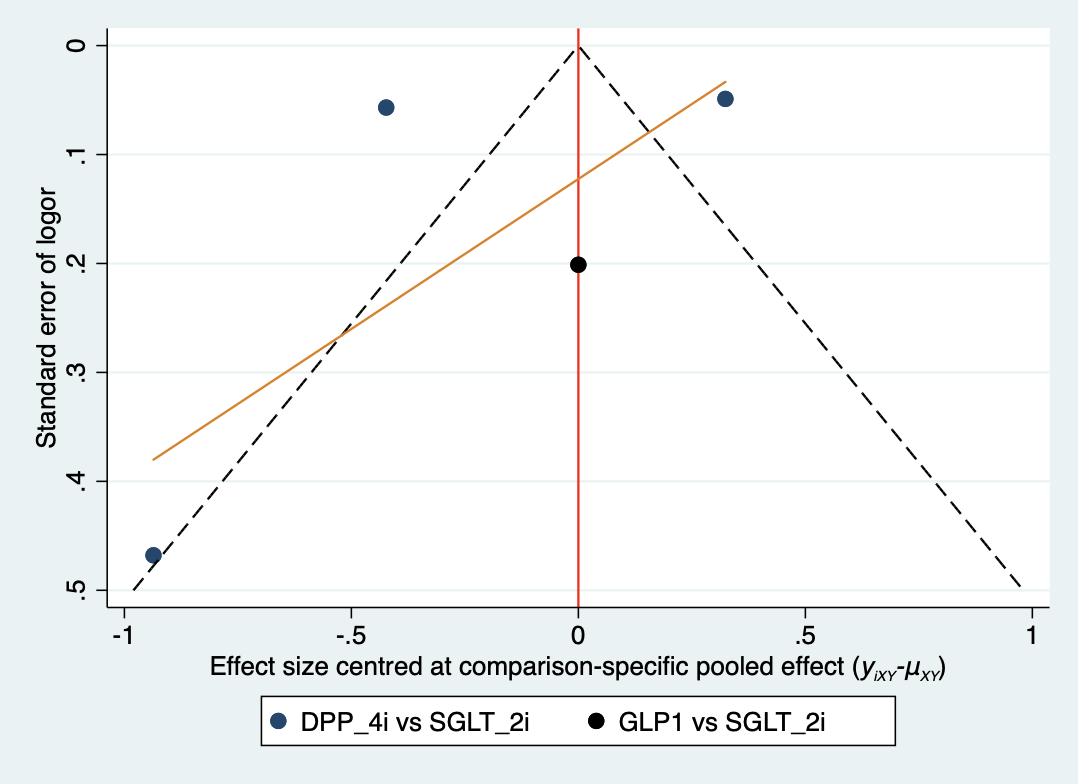


figure S5 Funnel plot of Alzheimer dementia


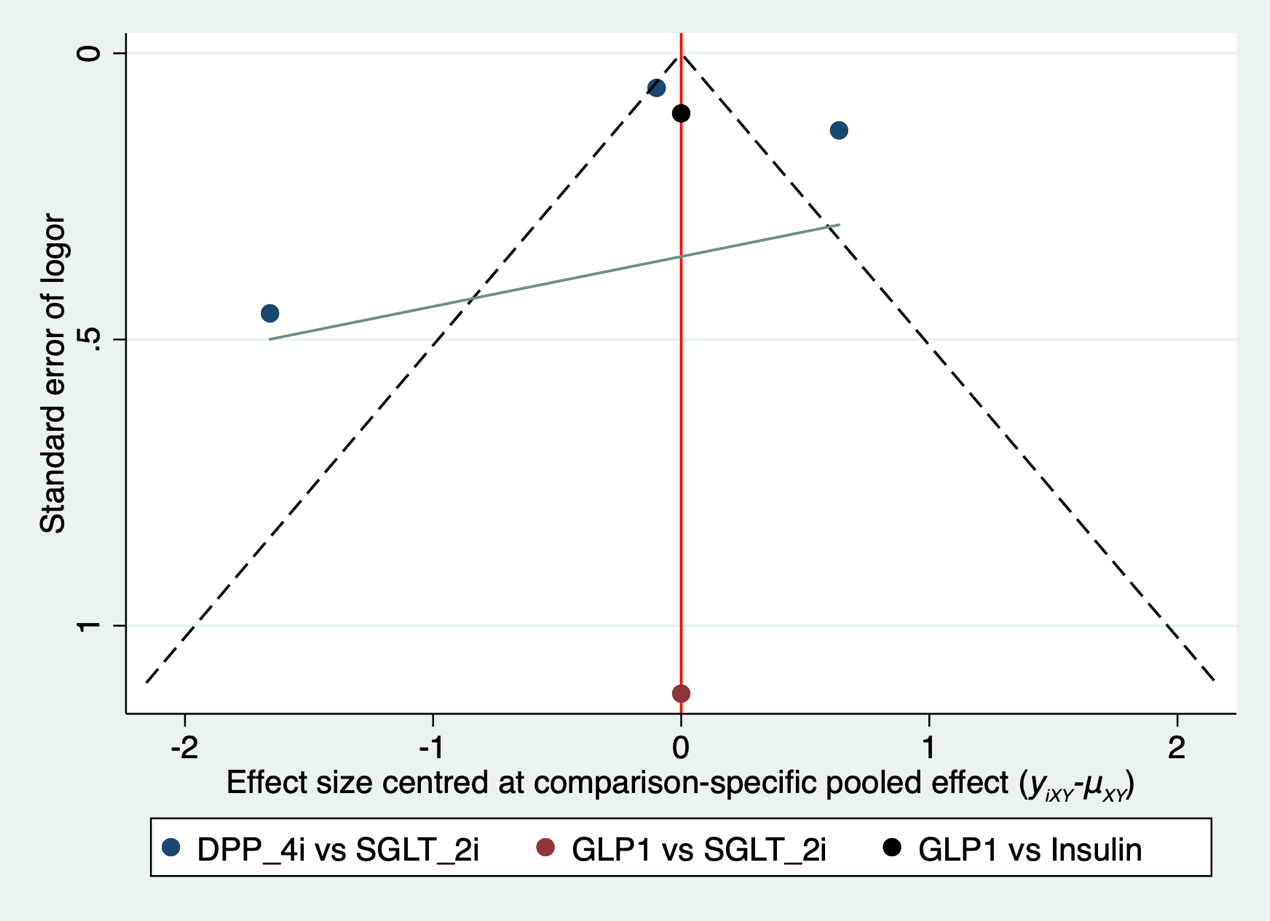


figure S5 Funnel plot of Vascular dementia
